# Epitope Spreading and the Efficacy of Immune Checkpoint Inhibition in Cancer

**DOI:** 10.23937/2643-4563/1710029

**Published:** 2021-05-28

**Authors:** W. Robert Liu, David E. Fisher

**Affiliations:** Cutaneous Biology Research Center, Department of Dermatology, Massachusetts General Hospital, Harvard Medical School, USA

## Abstract

Therapeutic antibodies that target immune checkpoints have revolutionized cancer therapy. While these checkpoints restrain T cell activation in response to antigen engagement, checkpoint inhibitors de-repress such tumor-associated T cells, and have generated major clinical responses in multiple tumor types. Nonetheless, the vast majority of cancers remain resistant to this therapeutic approach as currently deployed, either through intrinsic or acquired resistance mechanisms. One key question involves the identity of the tumor targets which effector T cells recognize. Tumor-specific mutant epitopes (often called neoantigens) represent a favored example, whose recognition has been demonstrated in certain contexts. While potentially helpful in identifying likely therapeutic opportunities (such as cancers harboring DNA repair defects), numerous cancers are relatively deficient in neoantigen loads. This commentary discusses the prospect that a phenomenon of “epitope spreading” may occur in certain high mutation contexts, giving rise to T cell responses against non-mutated/wild-type lineage proteins. Recent evidence is also discussed that suggests this mechanism may be exploited to purposely trigger epitope spreading and induce systemic tumor eradication in neoantigen-deficient cancers.

Immune checkpoint blockade, using anti-PD-1, anti-PD-L1, and anti-CTLA-4 antibodies, has transformed treatment of many kinds of cancer including melanoma. However, durable clinical response with significant tumor regression is elicited in only a small subset of cancer patients. Most cancer patients do not respond to immune checkpoint blockade at all (primary resistance); while other patients respond initially, they develop resistance over time and the cancers ultimately relapse (acquired resistance) [1].

Central questions in the field of immune checkpoint blockade for cancer treatment are: (1) Why do the majority of cancer patients’ tumors resist checkpoint blockade immunotherapy? What are the resistance mechanisms? How can we improve treatment efficacy? (2) How do cytotoxic T cells (CD8^+^ T cells) distinguish between normal cells and cancer cells for those patients who respond positively to immune checkpoint blockade? (3) How can toxicity associated with checkpoint blockade immunotherapy be understood, minimized, and/or eliminated? Answering these questions will not only provide unprecedented mechanistic insights into cancer immunology, but also remove barriers for improvements to checkpoint blockade immunotherapy.

In a study published in a recent issue of *Science Translational Medicine*, Lo, *et al*. [2] aimed to advance the field by offering key clues to some of the above long-standing questions, as discussed below:

## What antigenic determinants (epitopes) are responsible for tumor recognition during checkpoint blockade therapy in melanoma patients with low neoantigen loads?

Better responses to immune checkpoint blockade in patients with melanoma have been suggested to be associated with higher neoantigen loads, which are proteins mutated selectively in the cancer cells, many derived from UV radiation in the case of melanoma. Neoantigens on cancer cells may be recognized as “foreign” proteins and attacked by cytotoxic T cells [3]. Yet, some patients with melanomas carrying relatively low neoantigen loads still have excellent responses to immune checkpoint therapy.

Lo, *et al.* [2] hypothesized that immune responses against wild-type melanocyte antigens may contribute to efficacy of anti-CTLA-4 and anti-PD-1 checkpoint immunotherapies on the basis of the following evidence. First, employing gene set enrichment analysis (GSEA) of RNA-sequencing profiles of melanoma patients with comparably low neoantigen loads, the authors discovered that multiple pigmentation-related gene sets were enriched in ipilimumab responders compared with nonresponders. Second, the GO:0043473 pigmentation gene set was enriched in low neoantigen ipilimumab responders compared to nonresponders. Third, the authors observed similar results for nivolumab or pembrolizumab responders in additional cohorts of patients with melanoma. Lastly, the GO:0043473 pigmentation gene set contains many melanocyte differentiation antigens expressed in both melanoma cells and normal melanocytes.

The authors further supported this hypothesis by flow cytometry, demonstrating that anti-PD-1 treatment in HLA-A02^+^ patients with melanoma led to greater expansion of melanocyte antigen MART-1-specific CD8^+^ T cells in responders than in nonresponders. Thus, higher expression of MART-1 on melanoma cells suggests that wild-type lineage antigens may be targeted in low neoantigen tumors exhibiting good responses to anti-PD-1 immunotherapy.

These results might also help to explain why success of immune checkpoint blockade therapy for melanoma in the clinic is often accompanied by a side effect of autoimmune vitiligo, which is a skin disorder characterized by patches of the skin losing their pigment due to destruction of melanocytes (pigment cells). It is plausible that the CD8^+^ T cells also recognize MART-1 protein expressed on normal melanocytes and destroy clusters of them. Interestingly, the authors observed that only 5 out of the 13 responders developed vitiligo.

## Can somatic mutations in melanoma affect epitope spreading to wild-type melanocyte antigens after immune checkpoint blockade?

To understand this question, the authors utilized a mouse melanoma cell line containing BRAF (V600E) and PTEN homozygous loss (“parental D4M.3A.3”) but no UV-exposure or corresponding UV mutational load. They generated additional cell lines (D4M-UV2 and D4M-UV3) by irradiating the parental line with UV, followed by single cell cloning and exome sequencing of the derivative lines, which indeed harbored significant genomic UV mutational loads in the same general range as human cutaneous melanomas. Parental D4M.3A.3 and the UV2 line were implanted into syngeneic C57BL/6 mice and the corresponding tumors were then treated with anti-PD1. The authors found that after anti-PD1 treatment, mice bearing UV2 melanomas had higher gp100-specific CD8^+^ T cells compared with parental D4M.3A.3 melanomas. Glycoprotein 100 (gp100) is a wild-type melanocyte and melanoma lineage antigen. Immunohistochemistry results demonstrated that UV2 melanomas had higher tumor-infiltrating T cells compared with parental D4M.3A.3 melanomas. These results were corroborated by flow cytometry, showing that UV2 melanomas contained higher number of CD8^+^ T cells and greater immune cytolytic activity compared with parental D4M.3A.3 melanomas.

Conventional wisdom would predict that immune checkpoint blockade for melanomas with more neoantigens would exhibit expansion of mainly neoantigen-specific tumor infiltrating T cells. Interestingly, after anti-PD-1 treatment, melanomas with higher mutational loads exhibited a significant increase in tumor infiltrating T cells targeting the wild-type melanocyte antigen gp100. These results suggest that epitope spreading to wild-type lineage self-antigens was enhanced by the pre-existing higher neoantigen burden. Additionally, the authors demonstrated that mice cured from UV-mutated melanomas by anti-PD-1 were capable of resisting challenges by the parental melanoma which lacks UV mutations. This observation demonstrated the functional importance of epitope spreading and anti-tumor properties of lineage directed immunity against wild-type antigens.

## Can combined treatments improve anti-PD-1 immunotherapy efficacy in neoantigen-deficient tumors?

As described above, the parental D4M.3A.3 melanoma cell line exhibits a poor response to immune checkpoint inhibitors and thus serves as an *in vivo* model of neoantigen-deficient, poorly inflamed, and immunotherapy-resistant melanoma. Since epitope spreading was capable of triggering functional immunity against the neoantigen-poor parental melanoma, the authors tested whether non-mutational pro-inflammatory treatments might be capable of inducing epitope spreading towards wild-type melanocyte antigens on melanomas after anti-PD-1 treatment. To this end, they utilized a model consisting of the parental (neoantigen-poor) melanoma engrafted as two tumors on the flanks of syngeneic mice. They then treated one of the two/bilateral tumors with the triple combination of (1) ablative fractional photothermolysis (aFP), which is a cosmetic carbon dioxide laser producing nanoscale columns of tumor ablation over less than 5% of the tumor and aiming to induce tumor inflammation unilaterally; (2) topical imiquimod, a Toll-like receptor 7 (TLR7) agonist which activates innate immune signaling; and (3) anti-PD-1. Quadruple treatments (aFP + imiquimod + anti-PD-1 + anti-CTLA-4) were also tested and showed the strongest responses, resulting in complete regression of bilateral melanomas in 75% of mice with parental D4M.3A.3 melanomas. Triple treatments (aFP + imiquimod + anti-PD-1), any combination of double treatments, and single treatment induced complete bilateral regression of melanomas in 50%, 10%, and 0% of mice with D4M.3A.3 melanomas, respectively. One in 24 evaluable mice with complete response against melanoma developed vitiligo and none exhibited signs of gross toxicity. Importantly, these combination treatments were delivered locally to one of two tumors, yet they induced epitope spreading and complete regressions both locally as well as against contralateral tumors, thus demonstrating systemic immune activation.

## What is the underlying mechanism for improving anti-PD-1 therapeutic efficacy by aFP and imiquimod?

The authors performed RNA-sequencing as well as extensive immunophenotyping by flow cytometry to decipher molecular mechanisms underlying triple therapy (aFP + imiquimod + anti-PD-1) responses against melanomas with low mutational burden. The data suggest that: (1) Imiquimod enhances antigen presentation and activates the T cell compartment; (2) Triple therapy efficacy requires cross-priming of antitumor CD8^+^ T cells; (3) Triple therapy dramatically increases the number of gp100 tetramer-positive CD8^+^ tumor infiltrating lymphocytes; and (4) Triple therapy modulates expression of genes associated with immune signaling cascades and pigmentation. These data suggest that a cosmetic laser (aFP) plus TLR activation dramatically produces pro-inflammatory effects that enhance immune attack on wild-type melanocyte antigens on melanomas, resulting in improved anti-PD-1 immunotherapy efficacy. Similar efficacy was also observed for this combination treatment when applied to another poorly immunogenic tumor, pancreatic adenocarcinoma. Lo, *et al.* [2] thus provided evidence suggesting that low anti-PD-1 therapeutic efficacy for cancers containing low mutational loads might be improved by non-mutational strategies, such as physical or chemical means (aFP plus imiquimod treatment) that induce inflammatory responses.

## Conclusion

Mechanistically, either UV-irradiation or aFP plus imiquimod treatment can improve anti-PD-1 therapeutic efficacy in poorly immunogenic melanomas by expansion of CD8^+^ T cells capable of recognizing wild-type melanocyte antigens and boosting immune response. In addition to elucidating a mechanism likely underlying much antitumor efficacy by immune checkpoint inhibitors, these data also suggest new treatment opportunity for cancer harboring low neoantigen loads.

## Perspectives

Further studies are required to shed light on the following important questions raised by this study: (1) Can T cell populations specific for melanocyte antigens MART-1, tyrosinase, and other wild-type melanocyte antigens be used as biomarkers for monitoring responses to immune checkpoint blockade? (2) What comprehensive set of melanocyte antigens is targeted by the immune response after combinatorial immunotherapy? (3) What are fundamental principles or general rules/approaches for designing further combinatorial pro-inflammatory approaches that may enhance cancer immunotherapy? In which contexts will such an approach become detrimental due to elicitation of autoimmune responses? Decoding these questions will not only advance fundamental immunology and cancer biology, but also provide a roadmap for improving cancer immunotherapy using immune checkpoint blockade.
